# Interferon-gamma regulates inflammatory cell death by targeting necroptosis in experimental autoimmune arthritis

**DOI:** 10.1038/s41598-017-09767-0

**Published:** 2017-08-31

**Authors:** Seung Hoon Lee, Ji ye Kwon, Se-Young Kim, KyoungAh Jung, Mi-La Cho

**Affiliations:** 1 The Rheumatism Research Center, Catholic Research Institute of Medical Science, College of Medicine, The Catholic University of Korea, Seoul, South Korea; 2 Impact Biotech, Seoul, 137–040 South Korea; 3 Laboratory of Immune Network, Conversant Research Consortium in Immunologic disease, College of Medicine, The Catholic University of Korea, Seoul, South Korea

## Abstract

Interferon γ (IFN-γ) induces an inflammatory response and apoptotic cell death. Rheumatoid arthritis (RA) is a systemic inflammatory disease associated with increased levels of inflammatory mediators, including tumour necrosis factor α (TNF-α) and T helper (Th) 17 cells, and downregulation of apoptosis of inflammatory cells. We hypothesized that IFN-γ would reduce inflammatory cell death *in vitro* and that loss of IFN-γ would aggravate inflammation *in vivo*. IFN-γ downregulated necroptosis and the expression of cellular FLICE-like inhibitory protein (cFLIP_L_) and mixed lineage kinase domain-like (MLKL). However, loss of IFN-γ promoted the production of cFLIP_L_ and MLKL, and necroptosis. IFN-γ deficiency increased Th17 cell number and upregulated the expression of IL-17 and TNF-α. Expression of MLKL, receptor interacting protein kinase (RIPK)1, and RIPK3 was increased in the joints of mice with collagen-induced arthritis (CIA). Compared with wild-type mice with CIA, IFN-γ^−/−^ CIA mice showed exacerbation of cartilage damage and joint inflammation, and acceleration of MLKL, RIPK1, and RIPK3 production in the joints. IFN-γ deficiency induced the activation of signal transducer and activator of transcription 3. These results suggest that IFN-γ regulates inflammatory cell death and may have potential for use in the treatment of RA.

## Introduction

Necroptosis is defined as inflammatory cell death, which is a tightly programmed form of necrosis that can occur in a programmed manner under certain circumstances such as a massive inflammatory response^1, 2^. Cells undergo necroptosis rupture, which causes their contents to leak into the extracellular space. This leakage caused by cell rupture differs from the processes caused by apoptosis and aggravates the inflammatory response^2, 3^. Necroptosis plays a significant role in various pathophysiological states including systemic inflammatory response syndrome^4, 5^.

Several molecules are involved in necroptosis. Tumour necrosis factor α (TNF-α) triggers necroptosis in response to tissue injury and inflammation^6, 7^. Mixed lineage kinase domain-like (MLKL), receptor interacting protein kinase (RIPK1), and RIPK3 perform an important role in necroptosis. RIPK1 and RIPK3 form necrosomes and phosphorylate MLKL, and necrosomes induce necroptosis^8^. FLICE-like inhibitory protein (cFLIP_L_) has recently been shown to be involved in the increased expression of MLKL and necroptosis stimulated by TNF-α^9^.

Rheumatoid arthritis (RA) is a progressive autoimmune polyarthritis that is clinically characterized by systemic inflammation. RA can cause infiltration of inflammatory cells into the affected joints and chronic cartilage destruction^10^. The pathogenesis of RA is complicated and involves cell death and the actions of proinflammatory cytokines. It has been suggested that upregulation of proinflammatory cytokines is associated with the pathogenesis of RA^11^. However, apoptosis is decreased in RA patients. It is thought that deficient apoptosis in inflammatory cells may be involved in the pathogenesis of RA^12^.

The cytokine interferon γ (IFN-γ) is involved in cell death and the inflammatory response. IFN-γ ameliorates autoimmune disorders by suppressing the inflammatory response^13^. IFN-γ attenuates the differentiation of T helper (Th) 17 cells and osteoclasts, whereas loss of IFN-γ has a protective effect in collagen-induced arthritis (CIA)^14^. IFN-γ also increases the expression of apoptotic mediators^15, 16^.

We hypothesized that IFN-γ is involved in the pathogenesis of RA. The present investigation was conducted to determine whether IFN-γ has protective effects in experimental autoimmune arthritis by regulating necroptosis. We examined the effects of IFN-γ on necroptosis *in vitro* and measured the expression of molecular markers of necroptosis in an animal model of RA. We also analysed the progression of Th17 differentiation in IFN-γ-knockout mice.

## Results

### Loss of IFN-γ aggravated necroptosis and mitochondrial dysfunction

**Figure 1:**
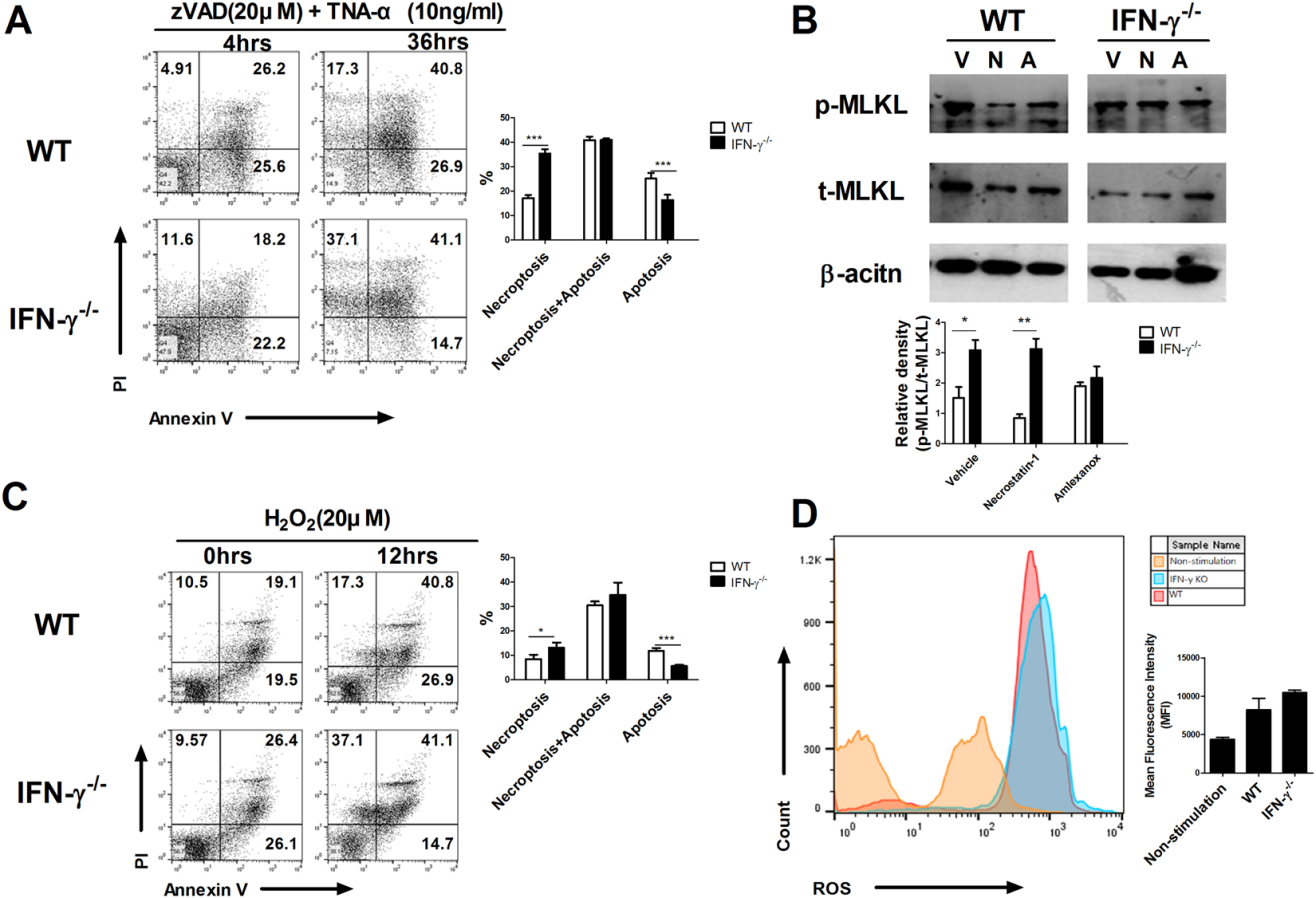
IFN-γ deficiency aggravated necroptosis. (**A**) The expression of PI and annexin V in splenocytes from WT and IFN-γ-knockout mice was induced by zVAD and TNF-α, and analysed by flow cytometry. The values represent the percentage of cells in each region (PI^+^ annexin V^−^: necroptosis, PI^+^ annexin V^+^: necroptosis + apoptosis, PI^−^ annexin V^+^: apoptosis). (**B**) Splenocytes of WT and IFN-γ-knockout mice were pretreated with vehicle (DMSO), necrostatin-1 (10 μM), or amlexanox (10 μM) for 2 hours, and then stimulated with zVAD and TNF-α for 1 hour. The cells were then prepared for determination of p-MLKL and t-MLKL expression. The level of p-MLKL is expressed as a relative ratio and t-MLKL was used to normalize the data. (**C**) Expression of PI and annexin V in splenocytes from WT and IFN-γ-knockout mice was induced by H_2_O_2_ and analysed by flow cytometry. (**D**) Expression of ROS in splenocytes from WT and IFN-γ-knockout mice was induced by H_2_O_2_ for 5 hours and analysed by flow cytometry.

We induced necroptosis in splenocytes obtained from wild-type (WT) and IFN-γ-knockout mice using zVAD and TNF-α. IFN-γ significantly increased the percentage of propidium iodide (PI)-positive cells at 36 hours compared with WT mice. By contrast, the percentage of annexin V-positive cells was decreased significantly in IFN-γ-knockout mice at 36 hours (Fig. 1A).

Because MLKL is a key marker of necroptosis related to RIPK1 activation^2^ and TANK-binding kinase 1 (TBK1) can regulate cell survival^17^, we measured MLKL expression in cells pretreated with necrostatin-1 or amlexanox. The expression of phosphorylated MLKL (p-MLKL) was higher in splenocytes from IFN-γ-knockout mice exposed to vehicle, necrostatin-1, or amlexanox compared with cells from WT mice (Fig. 1B). The percentage of PI-positive cells was significantly higher and the percentage of annexin V-positive cells was significantly lower in splenocytes from IFN-γ-knockout mice stimulated by H_2_O_2_ compared with those from WT mice (Fig. 1C). The intracellular level of reactive oxygen species (ROS) was also higher in splenocytes from IFN-γ-knockout mice than in cells from WT mice (Fig. 1D).

Because JC-1 exists as aggregates or monomers depending on the mitochondrial membrane potential in the normal state, we measured JC-1 fluorescence intensity. The number of red-fluorescing JC-1 aggregates was lower and that of JC-1-fluorescing green monomers was higher in splenocytes from IFN-γ-knockout mice compared with cells from WT mice. Quantitative analysis of the red–green fluorescence indicated that IFN-γ deficiency increased the intensity of green fluorescence (Supplementary Fig. 1A,B). These results suggest that loss of IFN-γ exacerbates necroptosis.

### IFN-γ reduced necroptosis and inhibited the expression of MLKL and cFLIP_L_

**Figure 2:**
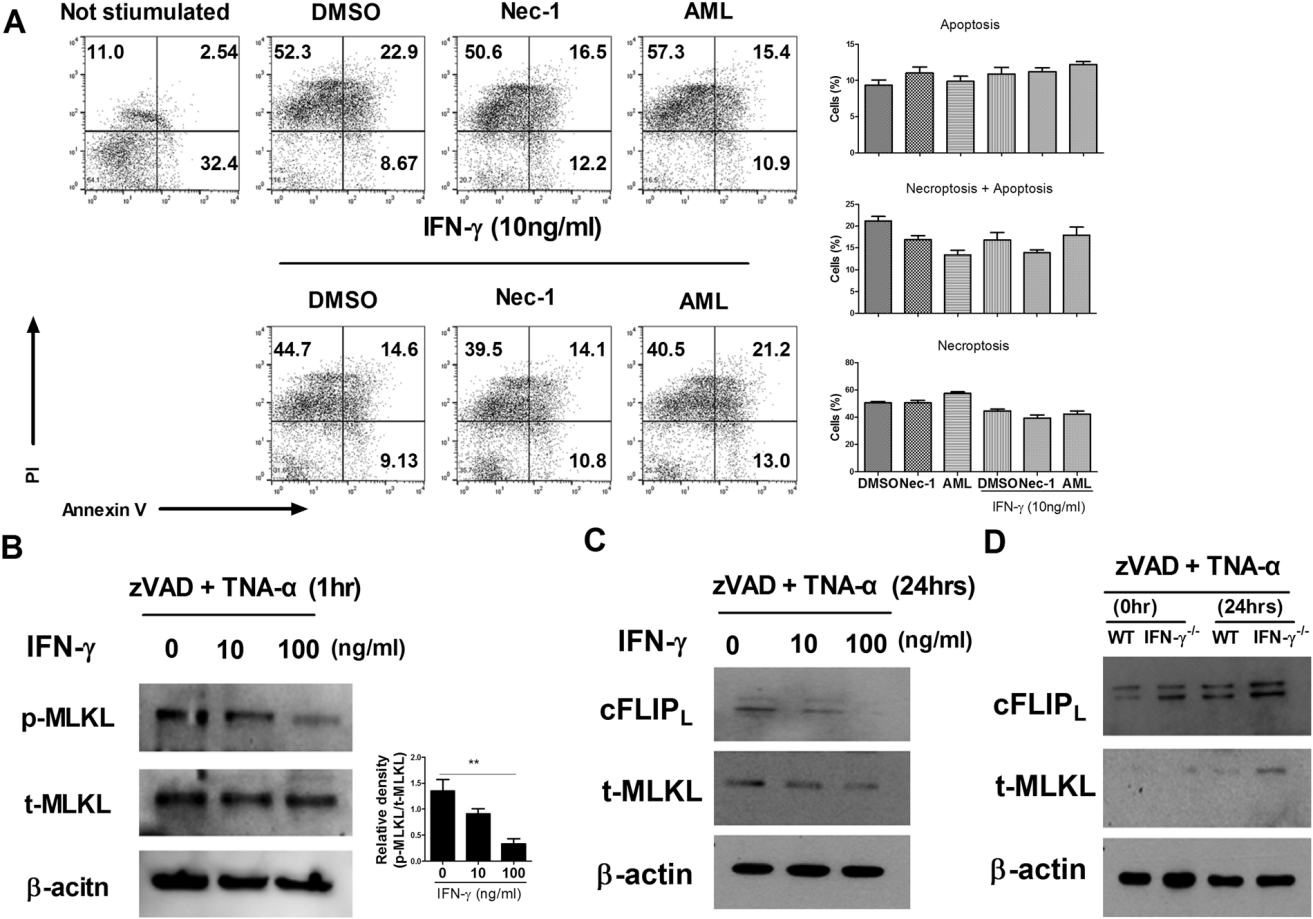
IFN-γ treatment downregulated necroptosis. (**A**) Expression of PI and annexin V in splenocytes from normal mice was induced by zVAD and TNF-α, and analysed by flow cytometry. The cells were pretreated with vehicle (DMSO), necrostatin-1 (10 μM), or amlexanox (10 μM) for 2 hours. (**B**) Normal mouse splenocytes were pretreated with IFN-γ and zVAD for 2 h and then stimulated with TNF-α for 1 hour. The cells were then prepared for determination of cFLIP_L_ and MLKL expression. (**C**) Normal mouse splenocytes were pretreated with IFN-γ and zVAD for 2 hours and then stimulated with TNF-α for 24 hours. The cells were then prepared for determination of p-MLKL and t-MLKL expression. (**D**) Splenocytes of WT and IFN-γ-knockout mice were pretreated with vehicle (DMSO), necrostatin-1 (10 μM), or amlexanox (10 μM) for 2 hours. The cells were then prepared for determination of cFLIP_L_ and MLKL expression.

Normal mouse splenocytes were stimulated with zVAD and TNF-α for 36 hours. To investigate whether IFN-γ is involved in inflammatory cell death, we introduced necrostatin-1, a necroptosis inhibitor that blocks RIPK1 activation^4^, and amlexanox, a TBK1 inhibitor that suppresses inflammation^18^. The cells were pretreated with dimethyl sulfoxide (DMSO), necrostatin-1, amlexanox, or IFN-γ. Treatment with IFN-γ, but not necrostatin-1 or amlexanox, decreased the percentage of PI-positive cells (Fig. 2A). The expression of cFLIP_L_ and MLKL in splenocytes was downregulated by IFN-γ treatment (Fig. 2B). IFN-γ treatment also decreased p-MLKL expression in splenocytes (Fig. 2C). Because cFLIP_L_ has antiapoptotic activity and is related to Th1 cell differentiation and the IFN-γ level^19, 20^, we analysed the expression of cFLIP_L_ and MLKL. IFN-γ deficiency increased the expression of cFLIP_L_ and MLKL (Fig. 2D). These results suggest that IFN-γ can attenuate necroptosis and decrease the production of cFLIP_L_ and MLKL.

### IFN-γ deficiency exacerbated the inflammatory response and Th17 cell differentiation

**Figure 3:**
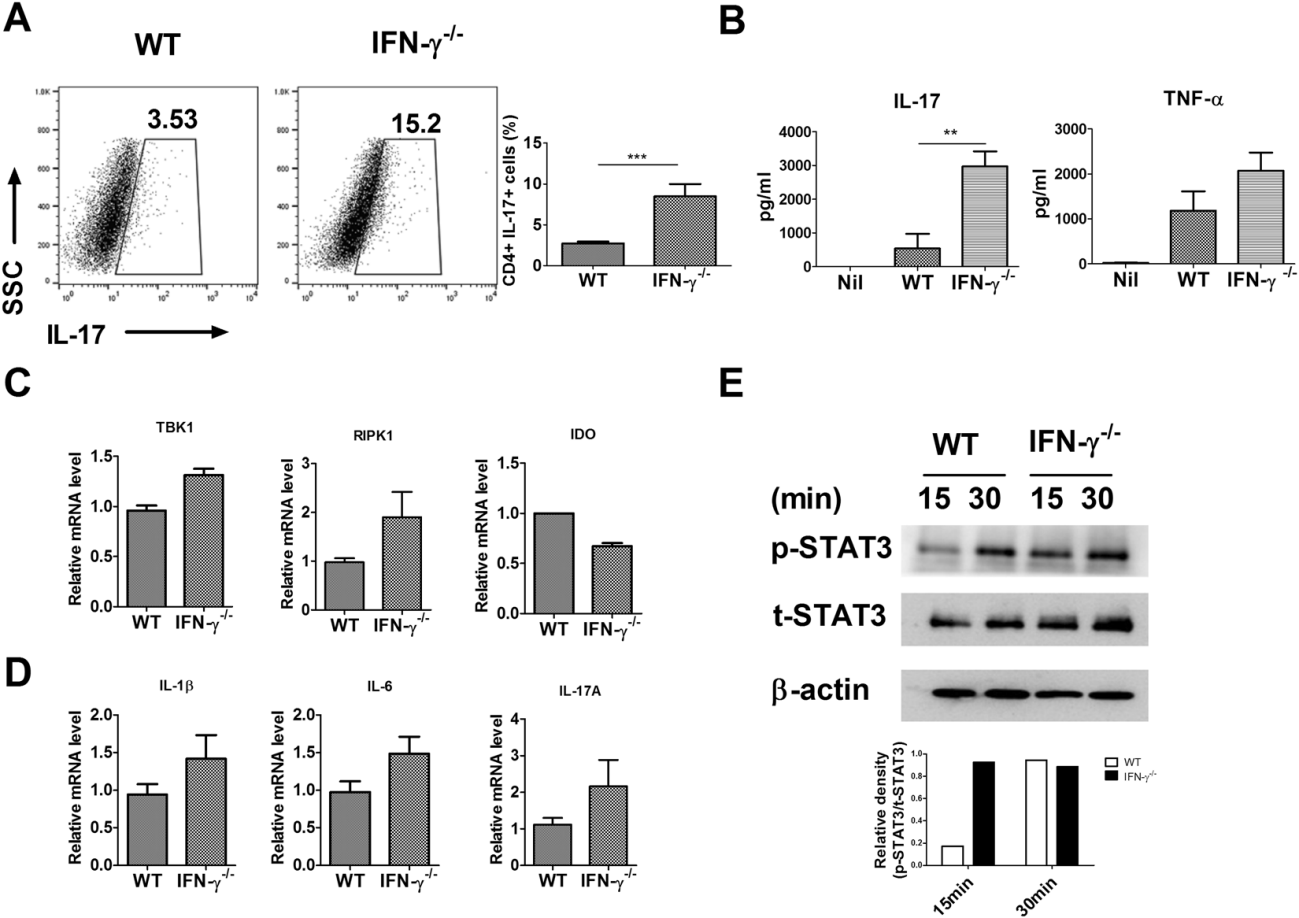
IFN-γ deficiency exacerbated the inflammatory response. (**A**) IL-17 expression in splenocytes from WT and IFN-γ-knockout mice incubated for 3 days under Th17 cell-polarizing conditions (stimulation only with anti-CD3 and anti-CD28 with TGF-β and IL-6) was measured by flow cytometry (n = 6). (**B**) IL-17 and TNF-α expression was analysed by ELISA using splenocytes from WT and IFN-γ-knockout mice cultured for 72 hours under Th17 cell-polarizing conditions (n = 6). (**C**) and (**D**) Relative mRNA expression was analysed by real-time PCR using splenocytes from WT and IFN-γ-knockout mice treated with TNF-α for 1 hour. (**E**) The level of p-STAT3 is expressed as a relative ratio and t-STAT3 was used to normalize the data. Splenocytes from WT and IFN-γ-knockout mice were stimulated under Th17-polarizing conditions. The cells were then prepared for determination of p-STAT3 and t-STAT3 expression. Data are expressed as mean ± standard deviation (SD) of three independent experiments (**P < 0.01, ***P < 0.005).

Because Th17 cells are involved in the pathogenesis of autoimmune arthritis^21, 22^, we analysed Th17 cell differentiation and the inflammatory response in splenocytes from WT and IFN-γ-knockout mice. IFN-γ deficiency stimulated the differentiation of Th17 cells and increased the expression of interleukin 17 (IL-17) and TNF-α (Fig. 3A,B). TBK1 and RIPK1 expression was also increased. The level of indoleamine 2,3-dioxygenase (IDO) mRNA was reduced (Fig. 3C), but the mRNA levels of proinflammatory cytokines were increased by IFN-γ deficiency (Fig. 3D). The expression of p-signal transducer and activator of transcription 3 (STAT3) was increased in splenocytes from IFN-γ-knockout mice (Fig. 3E). These findings suggest that loss of IFN-γ can stimulate the inflammatory response.

### Autoimmune arthritis increased the expression of key factors in necroptosis

**Figure 4:**
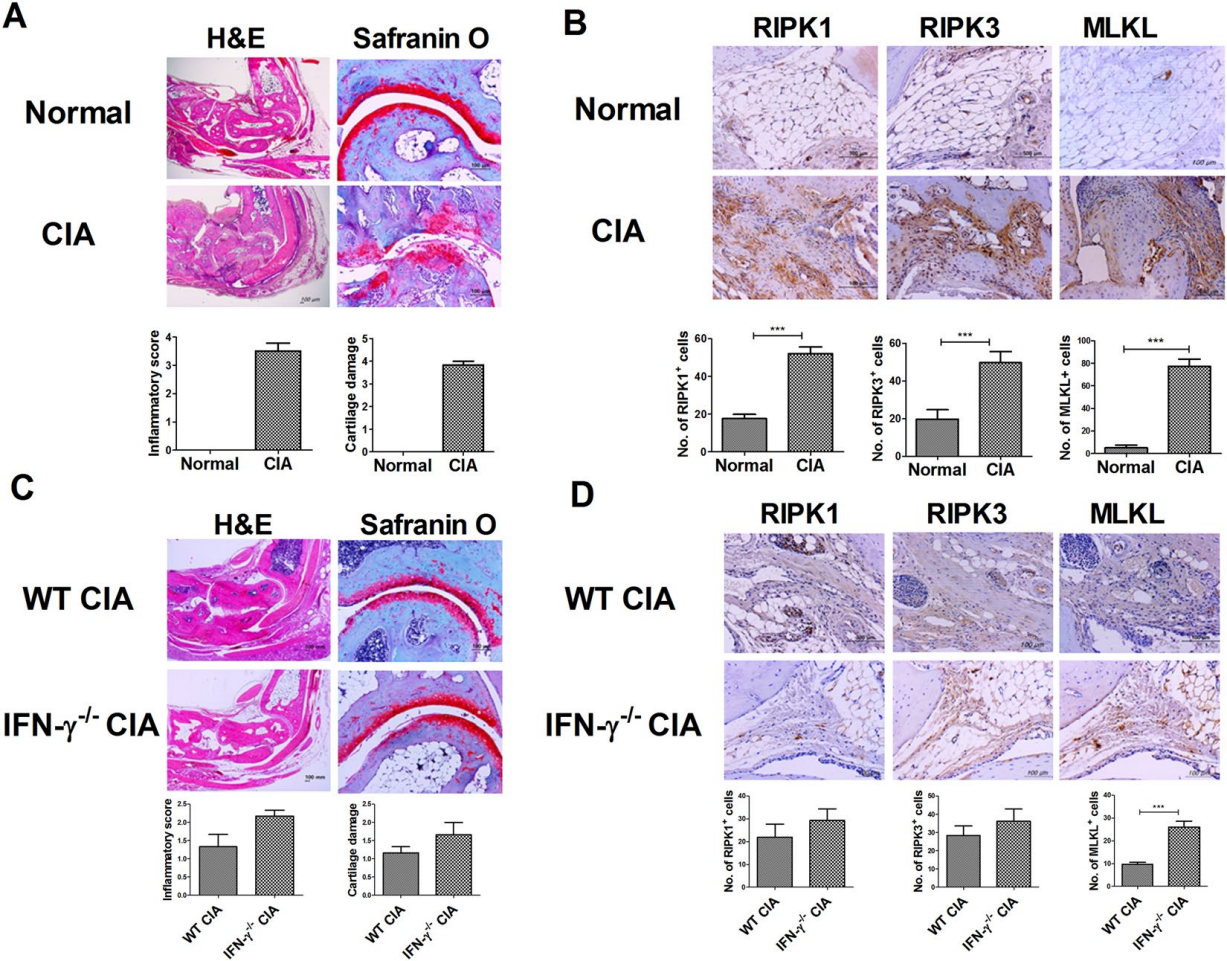
CIA induced necroptosis. (**A**) Joint tissues from normal and CIA mice were stained with haematoxylin and eosin, and Safranin O (n = 6). (**B**) Immunohistochemical visualization of RIPK1, RIPK3, and MLKL in the synovium of normal and CIA mice (n = 4). (**C**) Joint tissues from normal and WT and IFN-γ-knockout CIA mice were stained with haematoxylin and eosin, and Safranin O (n = 6). (**D**) Immunohistochemical visualization of RIPK1, RIPK3, and MLKL in the synovium of WT and IFN-γ-knockout CIA mice (n = 4). Data are expressed as mean ± SD (***P < 0.005).

We performed a histological analysis of joint tissue from naïve and CIA mice. CIA stimulated immune cell infiltration and cartilage damage (Fig. 4A). The expression of RIPK1, RIPK3, and MLKL was increased significantly in CIA mice (Fig. 4B). We also measured the expression of necroptosis factors in the synovium from WT and IFN-γ-knockout mice with CIA. We have previously reported that IFN-γ deficiency aggravates the inflammatory response and CIA progression^23^; however, in our previous work, we had not measured the expression of necroptosis factors in WT and IFN-γ-knockout mice with CIA. In the current study, IFN-γ deficiency showed trend increasing immune cell infiltration and cartilage damage (Fig. 4C). We also found trend increasing the expression of RIPK1 and RIPK3 in the synovium from IFN-γ-knockout mice with CIA. The expression of MLKL was increased significantly in the synovium from IFN-γ-knockout mice with CIA (Fig. 4D).

**Figure 5:**
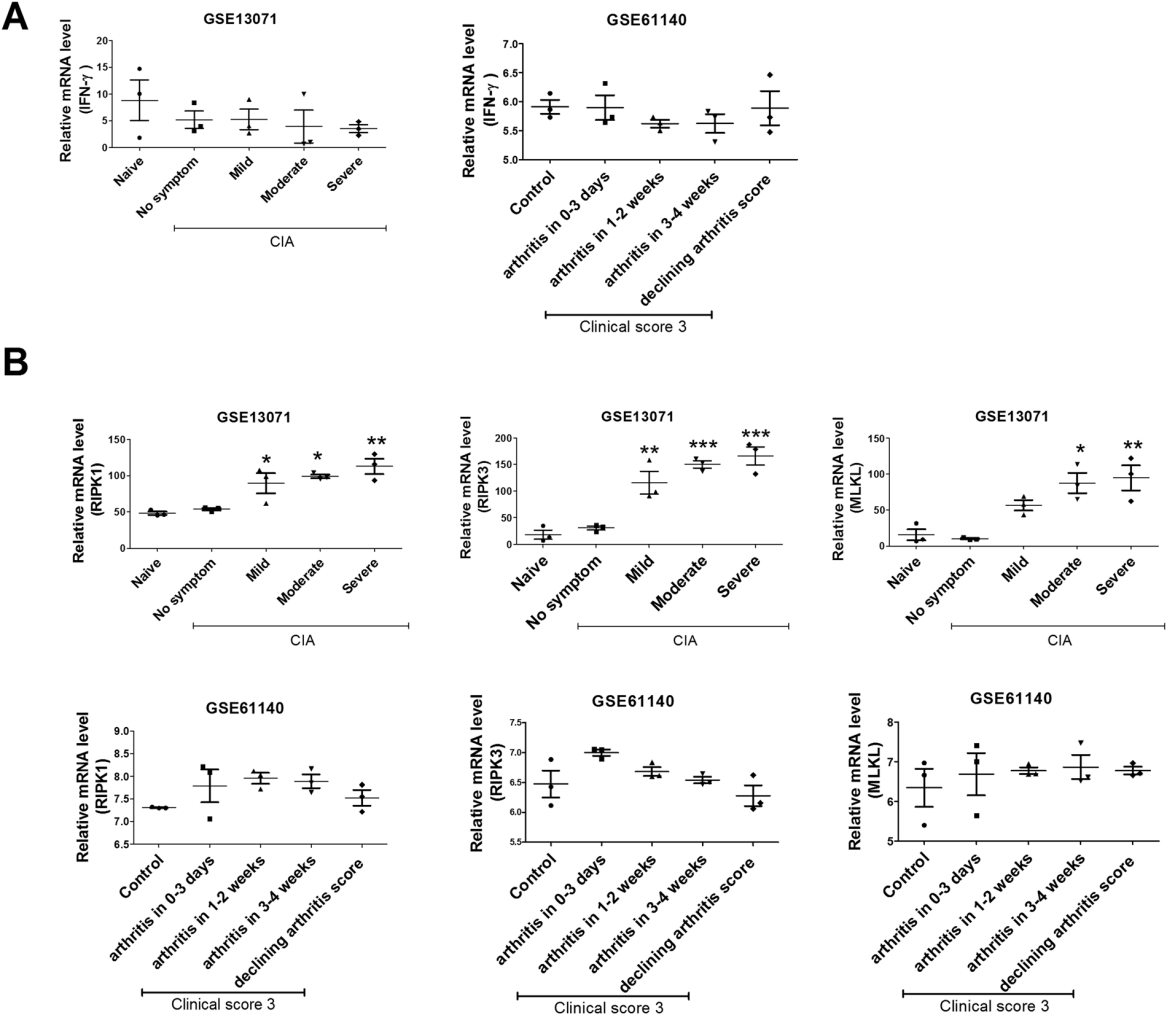
IFN-γ and necroptosis were involved in CIA. (**A**) and (**B**) IFN-γ, RIPK1, RIPK3, and MLKL expression in joint tissue of normal and CIA mice. Data are expressed as mean ± SD (*P < 0.05, **P < 0.03).

We analysed the relative IFN-γ mRNA levels in knee joint tissue of normal controls and CIA using information contained in the National Center for Biotechnology Information Gene Expression Omnibus database (GSE13071 and GSE61140). In our analysis, IFN-γ expression was lower than that in normal controls. In addition, the IFN-γ mRNA level decreased as the severity of CIA increased and recovered during CIA remission (Fig. 5A). The mRNA expression of RIPK1, RIPK3, and MLKL also increased with the severity of CIA: RIPK1, RIPK3, and MLKL expression increased for 0–3 days and declined during CIA remission (Fig. 5B).

We also analysed RIPK1, RIPK3, and MLKL expression using data on peripheral blood mononuclear cells contained in the database. RIPK3 and MLKL expression was higher in cells from patients with RA compared with those from healthy controls (Supplementary Fig. 2A). The GSE24742 database includes clinical and pathological data for 20 patients with RA. Rituximab treatment reduced RIPK1 and MLKL mRNA levels in synovium from patients with RA (Supplementary Fig. 2B). These results suggest that necroptosis may be involved in the pathogenesis of RA.

## Discussion

Although IFN-γ is involved in cell death and inflammation^24, 25^, there is no evidence that IFN-γ can attenuate necroptosis and little is known about the expression of necroptosis factors in CIA. Here, we investigated the role of IFN-γ in necroptosis and analysed the interaction between necroptosis and CIA.

The most remarkable result of this study was that IFN-γ regulated necroptosis. To our knowledge, this is the first report to show that IFN-γ attenuates necroptosis and decreases MLKL production. It has been suggested that necroptosis exacerbates inflammation and that inhibition of necroptosis attenuates the inflammatory response^2, 4^. MLKL also plays an essential role in autoimmune disease by inducing embryonic lethality^26^. Our findings indicate that IFN-γ can regulate necroptosis and inhibit MLKL production during necroptosis.

Although RA is characterized by exaggerated inflammation, the role of inflammation in the pathogenesis of RA is unclear. Necroptosis appears to mediate some immune responses and several inflammatory diseases^2^. Necroptosis mediators such as RIPK1 and MLKL are therapeutic targets in inflammatory diseases. RIPK1 is involved in the pathogenesis of multiple sclerosis, and inhibition of RIPK1 improves experimental autoimmune encephalomyelitis, an animal model of inflammation of the brain and spinal cord^27^. RIPK3 and MLKL expression increases significantly in inflamed tissue from patients with inflammatory bowel disease and allergic colitis^28^. We found that the expression of RIPK1, RIPK3, and MLKL was increased in the synovium of CIA mice. Moreover, the expression of necroptosis mediators was increased in joint tissue of CIA mice and the synovium of patients with RA. These results suggest that necroptosis may play an important role in the pathogenesis of inflammatory diseases such as RA.

STAT3 and Th17 play important roles in autoimmune arthritis. Inhibition of STAT3 activation improves the progression of experimental arthritis^21, 29^. Suppression of Th17 cell differentiation and inflammatory cytokines is also involved in the improvement of CIA^22^. We observed that loss of IFN-γ caused excessive Th17 cell differentiation and increased TNF-α and IL-17 expression by upregulating STAT3 activation. Moreover, IFN-γ suppresses IL-17 expression^30^. Our results suggest that IFN-γ inhibits the inflammatory response by downregulating Th17 cell differentiation and IL-17 production.

IFN-γ is a proinflammatory cytokine involved in Th1-driven immune responses. However, several studies have reported discrepant results regarding the role of IFN-γ in autoimmune disease. Deficiency of IFN-γ or the IFN-γ receptor increases the susceptibility to and severity of inflammatory disorders in experimental models^31–33^. Moreover, IFN-γ exerts a protective effect because of its anti-inflammatory activity and ability to downregulate cartilage damage in an experimental arthritis model^34, 35^. We found that the relative mRNA level of IFN-γ was decreased in the joint tissues of CIA mice but was increased in the synovium of RA patients. In addition, the production of RIPK1, RIPK3, and MLKL was significantly higher in the synovium of CIA mice compared with that of normal mice. We found that IFN-γ suppressed necroptosis and that loss of IFN-γ aggravated the inflammatory response. These findings suggest that IFN-γ may be effective as a treatment for RA by inhibiting necroptosis.

The expression of cFLIP_L_ is involved in IFN-γ production and cell death, including necroptosis. cFLIP_L_ is required for T cell proliferation^36^ and inhibits apoptosis and necroptosis^19, 37^. Additionally, loss of cFLIP_L_ makes T cells susceptible to apoptosis, and cFLIP_L_ expression negatively regulates Th1 cell differentiation and IFN-γ level^20^. Although the function of cFLIP_L_ in necroptosis is unclear, its overexpression leads to upregulation of MLKL and necroptosis mediated by TNF-α^9^. In the present investigation, we found that IFN-γ reduced the expression of cFLIP_L_, whereas IFN-γ deficiency increased cFLIP_L_ production. Our data suggest that IFN-γ regulates necroptosis by inhibiting MLKL and cFLIP_L_.

Accumulating evidence suggests that there is a need to rethink the proinflammatory function of IFN-γ in autoimmune diseases such as RA. IFN-γ treatment appears to have therapeutic activity and to improve RA progression, including swelling of an index joint^38^. We have also reported previously that IFN-γ deficiency exacerbates CIA development and joint inflammation, but we did not measure the levels of necroptosis mediators in CIA mice^23^. Initially, cell death was recognized as a result of chronic and excessive inflammation, but more recently, the concept of cell death has been classified into apoptosis, a weak inducer of inflammation, and necroptosis, a strong inducer of inflammation. Therefore, the IFN-γ activity associated with necroptosis suggests that IFN-γ may modulate necroptosis and improve RA progression by preventing or reducing inflammatory cell death.

## Methods

### Ethics statement

The Animal Care Committee of The Catholic University of Korea approved the experimental protocol, and all experimental procedures were carried out in accordance with the protocols approved by the Animal Research Ethics Committee at the Catholic University of Korea. All procedures performed followed the ethical guidelines for animal studies.

### Animals

Male C57BL/6, IFN-γ-knockout mice (8–10 weeks of age), and male DBA1/J mice (6–8 weeks of age) were maintained in groups of five in polycarbonate cages in a specific pathogen-free environment and were fed standard mouse chow (Ralston Purina, Gray Summit, MO, USA) and water *ad libitum*. IFN-γ-knockout mice were purchased from Jackson Laboratory, and DBA1/J mice were purchased from Orient Bio. All experimental procedures were examined and approved by the Animal Research Ethics Committee at the Catholic University of Korea.

### Type II collagen (CII) immunization and induction of CIA

CIA was induced in C57BL/6, IFN-γ-knockout, and DBA1/J mice (n = 5 per group). To induce CIA in C57BL/6 and IFN-γ-knockout mice, 200 μg/ml chicken CII (Chondrex Inc., Seattle, WA, USA) emulsified with complete Freund’s adjuvant (1:1 w/v) (Arthrogen-CIA, Redmond, WA, USA) was injected intradermally at the base of the tail. Three weeks later, a booster mixed with CII (100 μg/ml) emulsified with incomplete Freund’s adjuvant (1:1 w/v) (Difco, Detroit, MI, USA) was injected using the same protocol. DBA1/J mice were immunized intradermally into the base of the tail with 100 μg of chicken CII (Chondrex Inc., Redmond, WA, USA) in complete Freund’s adjuvant or incomplete Freund’s adjuvant (Chondrex, Inc.).

### Histological assessment of arthritis

Joint tissues obtained from each mouse were fixed in 10% formalin, decalcified in 10% ethylenediaminetetraacetic acid (EDTA), and embedded in paraffin wax for histological assessment. Haematoxylin and eosin-stained sections were scored for inflammation, pannus invasion, and bone and cartilage damage. The score was measured according to published criteria^39^.

### Cell preparation and culture

Mouse spleens were collected for cell preparation and washed twice with phosphate-buffered saline. The spleens were minced and the red blood cells were lysed with 0.83% ammonium chloride. The total splenocyte fraction was filtered through a cell strainer and centrifuged at 1,300 rpm at 4 °C for 5 min. The cells were stimulated with 0.5 μg/ml plate-bound anti-CD3 monoclonal antibody (mAb) (BD Biosciences, San Jose, CA, USA), 1 μg/ml soluble anti-CD28 mAb (BD Biosciences), 2 μg/ml anti-IFN-γ Ab (R&D Systems, Minneapolis, MN, USA), 2 μg/ml anti-IL-4 Ab (R&D Systems), 2 ng/ml recombinant transforming growth factor β (TGF-β) (R&D Systems), and 20 ng/ml recombinant IL-6 (R&D Systems) for 3 days to establish Th17 polarization. Peripheral blood mononuclear cells were isolated from buffy coats using Ficoll-Hypaque® (Amersham Biosciences, Pittsburgh, PA, USA) and density gradient centrifugation.

### Real-time polymerase chain reaction (PCR)

mRNA was extracted using TRI Reagent® (Molecular Research Center, Inc., Cincinnati, OH, USA) according to the manufacturer’s instructions. cDNA was synthesized using a SuperScript® Reverse Transcription system (TaKaRa). A LightCycler® 2.0 instrument (software version 4.0; Roche Diagnostics) was used for PCR amplification. All reactions were performed using the LightCycler® FastStart DNA Master SYBR® Green I Mix (TaKaRa) following the manufacturer’s instructions. The primer sequences used to amplify the mouse genes are listed in Supplementary Table 1. All mRNA levels were normalized to that of β-actin.

### Flow cytometry

Mononuclear cells were immunostained with various combinations of fluorescent antibodies against CD4, IL-17 (eBiosciences, San Diego, CA, USA), PI, and annexin V. Before intracellular staining, cells were restimulated for 4 h with phorbol myristate acetate (25 ng/ml) and ionomycin (250 ng/ml) in the presence of GolgiStop™ (BD Biosciences). Intracellular staining was performed using a kit (eBiosciences) following the manufacturer’s protocol. Flow cytometry was performed on a FACSCalibur™ flow cytometer (BD Biosciences).

### Enzyme-linked immunosorbent assay (ELISA)

Cytokine levels in culture supernatants were measured by sandwich ELISA. Antibodies directed against mouse TNF-α and IL-17 and biotinylated anti-mouse TNF-α and IL-17 (R&D Systems) were used as the capture and detection antibodies, respectively. The concentration of cytokines present in the test samples was determined from standard curves established with serial dilutions of recombinant TNF-α and IL-17 (R&D Systems). The absorbance was measured using an ELISA microplate reader at 405 nm (Molecular Devices, Sunnyvale, CA, USA).

### Immunohistochemistry

Immunohistochemistry was performed using a Vectastain® ABC Kit (Vector Laboratories). Tissues were first incubated with primary anti-RIPK1, anti-RIPK3, or anti-MLKL antibodies overnight at 4 °C. The primary antibody was detected with a biotinylated secondary antibody, the samples were incubated with streptavidin–peroxidase complex for 1 h, and 3,3′-diaminobenzidine chromogen was added to obtain a coloured product (Dako).

### Western blot analysis

Total proteins were extracted with lysis buffer containing 1% Nonidet P-40, phenylmethylsulfonyl fluoride, 2 mM sodium vanadate, 0.1% sodium deoxycholate, and a protease inhibitor mixture (Roche Applied Science, Mannheim, Germany). Proteins were loaded onto 10% polyacrylamide gels and subjected to sodium dodecyl sulfate-polyacrylamide gel electrophoresis, and the bands were transferred to nitrocellulose membranes (Invitrogen Life Technologies, Carlsbad, CA, USA). Membranes were blocked with 5% (w/v) non-fat milk in Tris-buffered saline with 0.1% Tween-20 for 1 hour at room temperature and incubated with antibodies to p-STAT3 Y705, t-STAT3, p-MLKL, t-MLKL (Cell Signaling Technologies), cFLIP_L_, and β-actin (Santa Cruz Biotechnology), or FLAG (Sigma-Aldrich) overnight at 4 °C. The membranes were then incubated with goat anti-mouse or goat anti-rabbit horseradish peroxidase-conjugated antibodies. Immunoreactivity was determined using enhanced chemiluminescence reagents (Amersham Biosciences, Piscataway, NJ, USA). ImageJ software was used for densitometric quantification of the immunoblots. The value of each band was normalized to that of t-MLKL or t-STAT3.

### Statistical analysis

The data were analysed using the nonparametric Mann–Whitney *U* test to compare two groups or one-way analysis of variance with Bonferroni’s *post hoc* test for multiple comparisons. GraphPad Prism® (ver. 5.01) was used for all analyses. A value of *P* < 0.05 was considered to indicate significance. Data are expressed as mean ± standard deviation (SD).

## Electronic supplementary material

Supplementary information

## References

[1] Galluzzi L (2015). Essential versus accessory aspects of cell death: recommendations of the NCCD 2015. Cell death and differentiation.

[2] Pasparakis M, Vandenabeele P (2015). Necroptosis and its role in inflammation. Nature.

[3] Vandenabeele P, Galluzzi L, Vanden Berghe T, Kroemer G (2010). Molecular mechanisms of necroptosis: an ordered cellular explosion. Nature reviews. Molecular cell biology.

[4] Linkermann A, Green DR (2014). Necroptosis. The New England journal of medicine.

[5] Vanden Berghe T, Linkermann A, Jouan-Lanhouet S, Walczak H, Vandenabeele P (2014). Regulated necrosis: the expanding network of non-apoptotic cell death pathways. Nature reviews. Molecular cell biology.

[6] He S (2009). Receptor interacting protein kinase-3 determines cellular necrotic response to TNF-alpha. Cell.

[7] Roychowdhury S, McMullen MR, Pisano SG, Liu X, Nagy LE (2013). Absence of receptor interacting protein kinase 3 prevents ethanol-induced liver injury. Hepatology.

[8] Zhao J (2012). Mixed lineage kinase domain-like is a key receptor interacting protein 3 downstream component of TNF-induced necrosis. Proceedings of the National Academy of Sciences of the United States of America.

[9] Shindo R, Yamazaki S, Ohmuraya M, Araki K, Nakano H (2016). Short form FLICE-inhibitory protein promotes TNFalpha-induced necroptosis in fibroblasts derived from CFLARs transgenic mice. Biochemical and biophysical research communications.

[10] Firestein GS (2003). Evolving concepts of rheumatoid arthritis. Nature.

[11] Smolen JS (2005). Pro-inflammatory cytokines in rheumatoid arthritis: pathogenetic and therapeutic aspects. Clinical reviews in allergy & immunology.

[12] Pope RM (2002). Apoptosis as a therapeutic tool in rheumatoid arthritis. Nature reviews. Immunology.

[13] Matthys P, Vermeire K, Heremans H, Billiau A (2000). The protective effect of IFN-gamma in experimental autoimmune diseases: a central role of mycobacterial adjuvant-induced myelopoiesis. Journal of leukocyte biology.

[14] Schurgers E, Billiau A, Matthys P (2011). Collagen-induced arthritis as an animal model for rheumatoid arthritis: focus on interferon-gamma. Journal of interferon & cytokine research: the official journal of the International Society for Interferon and Cytokine Research.

[15] Park SY (2004). IFN-gamma enhances TRAIL-induced apoptosis through IRF-1. European journal of biochemistry.

[16] Perez-Rodriguez R, Roncero C, Olivan AM, Gonzalez MP, Oset-Gasque MJ (2009). Signaling mechanisms of interferon gamma induced apoptosis in chromaffin cells: involvement of nNOS, iNOS, and NFkappaB. Journal of neurochemistry.

[17] Delhase M (2012). TANK-binding kinase 1 (TBK1) controls cell survival through PAI-2/serpinB2 and transglutaminase 2. Proceedings of the National Academy of Sciences of the United States of America.

[18] Reilly SM (2013). An inhibitor of the protein kinases TBK1 and IKK-varepsilon improves obesity-related metabolic dysfunctions in mice. Nature medicine.

[19] Ranjan K, Pathak C (2016). FADD regulates NF-kappaB activation and promotes ubiquitination of cFLIPL to induce apoptosis. Scientific reports.

[20] Kylaniemi MK, Kaukonen R, Myllyviita J, Rasool O, Lahesmaa R (2014). The regulation and role of c-FLIP in human Th cell differentiation. PloS one.

[21] Lee SH (2016). PTEN ameliorates autoimmune arthritis through down-regulating STAT3 activation with reciprocal balance of Th17 and Tregs. Scientific reports.

[22] Park MJ (2016). Overexpression of soluble RAGE in mesenchymal stem cells enhances their immunoregulatory potential for cellular therapy in autoimmune arthritis. Scientific reports.

[23] Lee J (2013). Interferon gamma suppresses collagen-induced arthritis by regulation of Th17 through the induction of indoleamine-2,3-deoxygenase. PloS one.

[24] Rakshit S (2014). Interferon-gamma induced cell death: Regulation and contributions of nitric oxide, cJun N-terminal kinase, reactive oxygen species and peroxynitrite. Biochimica et biophysica acta.

[25] Lu Y (2012). IFN-gamma and indoleamine 2,3-dioxygenase signaling between donor dendritic cells and T cells regulates graft versus host and graft versus leukemia activity. Blood.

[26] Alvarez-Diaz S (2016). The Pseudokinase MLKL and the Kinase RIPK3 Have Distinct Roles in Autoimmune Disease Caused by Loss of Death-Receptor-Induced Apoptosis. Immunity.

[27] Ofengeim D (2015). Activation of necroptosis in multiple sclerosis. Cell reports.

[28] Pierdomenico M (2014). Necroptosis is active in children with inflammatory bowel disease and contributes to heighten intestinal inflammation. The American journal of gastroenterology.

[29] Hayat F (2016). STX0119 ameliorates arthritis in SKG mice via inhibiting T helper 17. Tissue Engineering and Regenerative Medicine.

[30] Park H (2005). A distinct lineage of CD4 T cells regulates tissue inflammation by producing interleukin 17. Nature immunology.

[31] Jones LS (1997). IFN-gamma-deficient mice develop experimental autoimmune uveitis in the context of a deviant effector response. Journal of immunology.

[32] Ferber IA (1996). Mice with a disrupted IFN-gamma gene are susceptible to the induction of experimental autoimmune encephalomyelitis (EAE). Journal of immunology.

[33] Manoury-Schwartz B (1997). High susceptibility to collagen-induced arthritis in mice lacking IFN-gamma receptors. Journal of immunology.

[34] Page CE (2010). Interferon-gamma inhibits interleukin-1beta-induced matrix metalloproteinase production by synovial fibroblasts and protects articular cartilage in early arthritis. Arthritis research & therapy.

[35] Williams AS (2007). Interferon-gamma protects against the development of structural damage in experimental arthritis by regulating polymorphonuclear neutrophil influx into diseased joints. Arthritis and rheumatism.

[36] Zhang N, Hopkins K, He YW (2008). The long isoform of cellular FLIP is essential for T lymphocyte proliferation through an NF-kappaB-independent pathway. Journal of immunology.

[37] Oberst A (2011). Catalytic activity of the caspase-8-FLIP(L) complex inhibits RIPK3-dependent necrosis. Nature.

[38] Machold KP, Neumann K, Smolen JS (1992). Recombinant human interferon gamma in the treatment of rheumatoid arthritis: double blind placebo controlled study. Annals of the rheumatic diseases.

[39] Camps M (2005). Blockade of PI3Kgamma suppresses joint inflammation and damage in mouse models of rheumatoid arthritis. Nature medicine.

