# Supplementary material for: Interferon-gamma regulates inflammatory cell death by targeting necroptosis in experimental autoimmune arthritis
Source: Sci Rep. 2017 Aug 31;7:10133. doi: 10.1038/s41598-017-09767-0 (PMC5579272; doi:10.1038/s41598-017-09767-0)
Supplement: Supplementary file 1 — Supplementary information [file 41598_2017_9767_MOESM1_ESM.pdf]

Interferon-gamma regulates inflammatory cell death by targeting necroptosis in experimental autoimmune arthritis

Subtitle: IFN-γ regulates necroptosis

Seung Hoon Lee[1], Ji ye Kwon[1], Se-Young Kim[1], KyoungAh Jung[2], and Mi-La Cho[1, 3]

[1] The Rheumatism Research Center, Catholic Research Institute of Medical Science, The Catholic University of Korea, Seoul, South Korea

[2] Impact Biotech, Seoul, 137-040, South Korea

[3] Laboratory of Immune Network, Conversant Research Consortium in Immunologic disease, College of Medicine, The Catholic University of Korea

Correspondence:

Mi-La Cho, PhD, Rheumatism Research Center, Catholic Institutes of Medical Science, The Catholic University of Korea, 505 Banpo-dong, Seocho-gu, Seoul 137-040, Korea (South), Phone: 82-2-2258-7467; Fax: 82-2-599-4287; E-mail: iammila@catholic.ac.kr

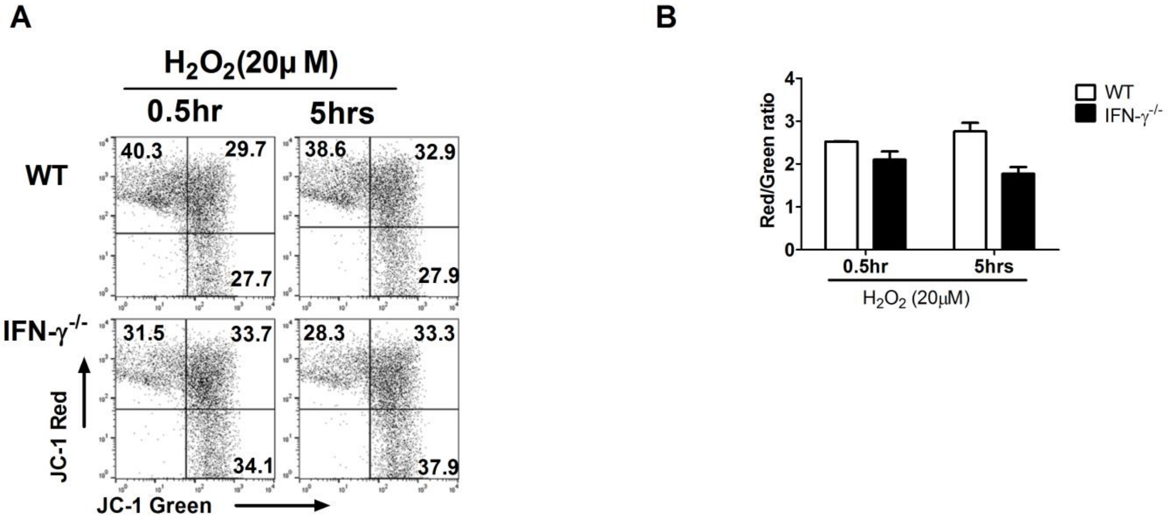

Supplementary figure 1. IFN-γ deficiency increases necroptosis and induces downregulation of mitochondrial membrane potential. (A and B) Mitochondrial membrane potential changes were analyzed by flow cytometry using JC-1. Fluorescence intensity shifted from the higher level to the lower one indicates the loss of mitochondrial membrane potential. Quantitative analysis of the green fluorescence (JC-1 monomer) showed that the NAC/AAP decreased green fluorescence in the absence or presence of Nec1 (right panel). Mitochondria depolarization is indicated by an increase in the red fluorescence intensity ratio. Data are presented as the mean ± SD (*P < 0.05, **P < 0.03).

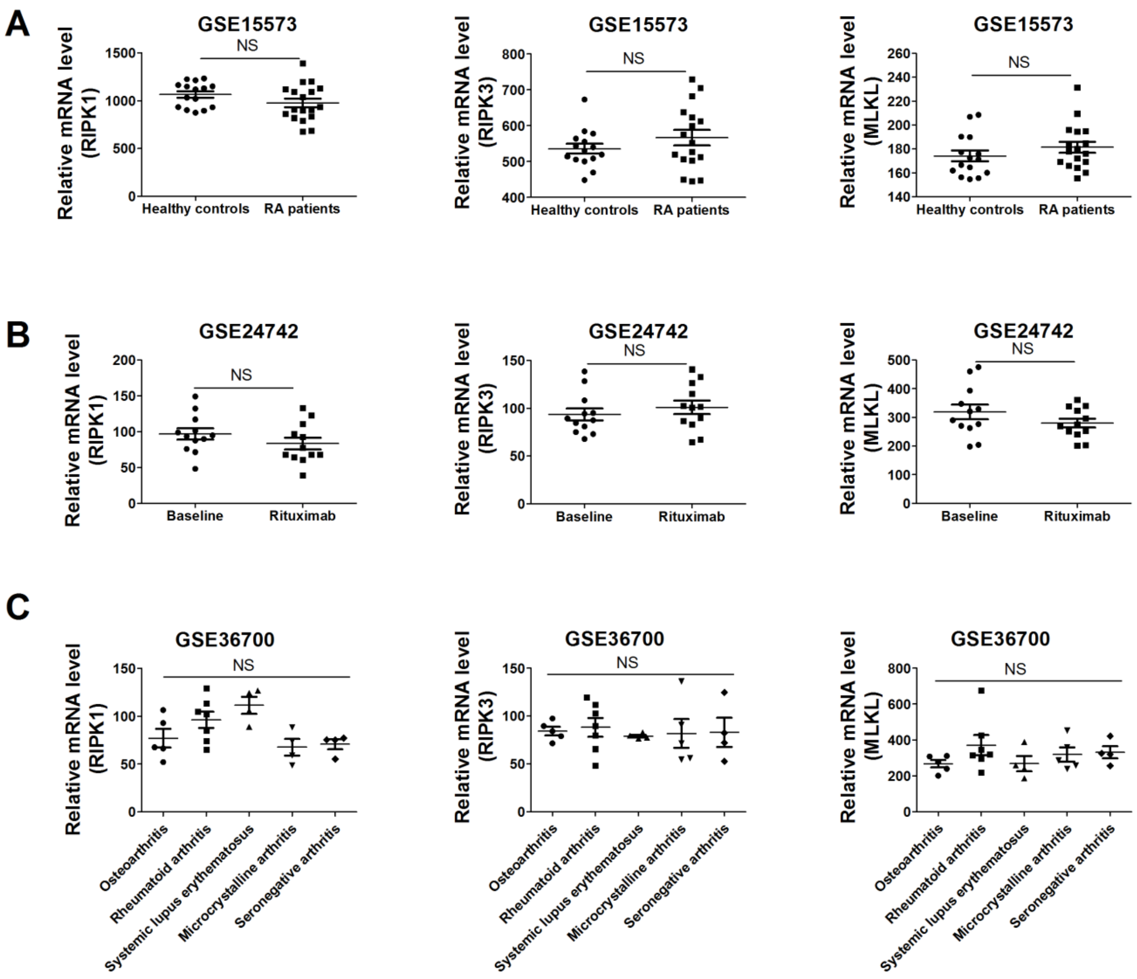

Supplementary figure 2. Rheumatoid arthritis increases necroptosis factors. (A) Gene expression of RIPK1, RIPK3 and MLKL in peripheral blood mononuclear cells of healthy controls and RA patients. (B) Gene expression of RIPK1, RIPK3 and MLKL in peripheral blood mononuclear cells of RA patients. (C) Gene expression of RIPK1, RIPK3 and MLKL in synovium of patients with several diseases including RA. Data are presented as the mean ± SD.

Gene	Sense primer (5'-->3)	Antisense primer (3'-->5')
IL-17A	CCT CAA AGC TCA GCG TGT CC	GAG CTC ACT TTT GCG CCA AG
IL-1β	GGA TGA GGA CAT GAG CAC ATT C	GGA AGA CAG GCT TGT GCT CTG A
IL-6	AAC GAT GAT GCA CTT GCA GAA A	TCT GAA GGA CTC TGG CTT TGT C
IDO	GAC GGA CTG AGA GGA CAC AG	GGC AGC ACC TTT CGA ACA TC
TBK1	GAC ATG CCT CTC TCC TGT AGT C	GGT GAA GCA CAT CAC TGG TCT C
RIPK1	CTG TTC CCT GTG CCC AAT AA	ATG ACT CTG AAG CTG TCC TTT C
β-actin	GAAATCGTGCGTGACATCAAAG	TGTAGTTTCATGGATGCCACAG

Supplementary Table 1. PCR primer sequence used in this study.
